# Sex Differences in Neural Activation to Facial Expressions Denoting Contempt and Disgust

**DOI:** 10.1371/journal.pone.0003622

**Published:** 2008-11-05

**Authors:** André Aleman, Marte Swart

**Affiliations:** 1 BCN Neuroimaging Center, University Medical Center Groningen, University of Groningen, Groningen, the Netherlands; 2 Department of Experimental Psychology, University of Utrecht, Utrecht, the Netherlands; James Cook University, Australia

## Abstract

The facial expression of contempt has been regarded to communicate feelings of moral superiority. Contempt is an emotion that is closely related to disgust, but in contrast to disgust, contempt is inherently interpersonal and hierarchical. The aim of this study was twofold. First, to investigate the hypothesis of preferential amygdala responses to contempt expressions versus disgust. Second, to investigate whether, at a neural level, men would respond stronger to biological signals of interpersonal superiority (e.g., contempt) than women. We performed an experiment using functional magnetic resonance imaging (fMRI), in which participants watched facial expressions of contempt and disgust in addition to neutral expressions. The faces were presented as distractors in an oddball task in which participants had to react to one target face. Facial expressions of contempt and disgust activated a network of brain regions, including prefrontal areas (superior, middle and medial prefrontal gyrus), anterior cingulate, insula, amygdala, parietal cortex, fusiform gyrus, occipital cortex, putamen and thalamus. Contemptuous faces did not elicit stronger amygdala activation than did disgusted expressions. To limit the number of statistical comparisons, we confined our analyses of sex differences to the frontal and temporal lobes. Men displayed stronger brain activation than women to facial expressions of contempt in the medial frontal gyrus, inferior frontal gyrus, and superior temporal gyrus. Conversely, women showed stronger neural responses than men to facial expressions of disgust. In addition, the effect of stimulus sex differed for men versus women. Specifically, women showed stronger responses to male contemptuous faces (as compared to female expressions), in the insula and middle frontal gyrus. Contempt has been conceptualized as signaling perceived moral violations of social hierarchy, whereas disgust would signal violations of physical purity. Thus, our results suggest a neural basis for sex differences in moral sensitivity regarding hierarchy on the one hand and physical purity on the other.

## Introduction

Contempt and disgust are closely related emotions, that have been considered to be “moral emotions”. According to Rozin et al. [1], contempt is a response to a violation of moral codes regarding disrespect, duty or hierarchy. These codes have been referred to as “ethics of community” [2]. Disgust, on the other hand, is a response to violations of physical purity, such as food and sex taboos. These codes have been referred to as “ethics of divinity” [2]. Both emotions involve rejection, disapproval and a degree of hostility. However, in contrast to disgust, which can concern inanimate objects, the expression of contempt inherently signals social, interpersonal information. In other words, it is always directed at a person. The expression of contempt bears upon social dominance, as it is directed downward in contrastive comparisons [3]. Thus, if a person expresses contempt towards another person, that person signals to regard himself as superior to the other, indicating that this universal facial expression [4], [5] may directly be linked with positioning in the social hierarchy. Disgust, on the other hand, can be induced by nonsocial stimuli such as eating bad food. Disgust can also be directed at persons, but will not bear upon the person perse, but rather on his behavior in violating sociocultural rules regarding physical purity.

The amygdala is a key structure of the emotional brain and has been regarded a socio-emotional relevance detector [6]. It has recently been suggested that contempt expressions will elicit stronger amygdala activation than disgust, due to the stronger social component of contempt [7]. In this study, we tested this hypothesis by using an oddball design that minimizes habituation of amygdala responses to faces.

Our second hypothesis concerned sex differences in activation to expressions of contempt versus disgust. The emotion of contempt may have a functional role in marking out and maintaining distinctions of rank and prestige [1], [8]. Men have been shown to be more sensitive than women to social dominance and hierarchy [9]. We hypothesized that, at a neural level, men would respond stronger to biological signals of interpersonal superiority than women.

During scanning with fMRI, participants (N = 16) viewed pictures of faces from a standard set, depicting expressions of contempt and disgust in addition to neutral expressions. The face stimuli were presented in a sequence of meaningless stimuli (random dot patterns) to prevent habituation of emotional brain structures (which may not respond anymore after repeated exposure to emotional stimuli). Thus, subjects were presented with random dot patterns (“standards”) interspersed with target stimuli (to which they had to react with a button press) and novel distractors (the face stimuli which were the actual stimuli of interest for our analyses). The standards were presented for 82% of trials, whereas the other stimuli (target face, neutral faces, disgusted faces and contemptuous faces) were presented for 2.33% of trials. The task for the subject was to press a response button whenever one specific male face (the target) was presented. This instruction ensured us that the subject paid attention to the stimuli and allowed for an objective verification.

## Results

**Figure 1:**
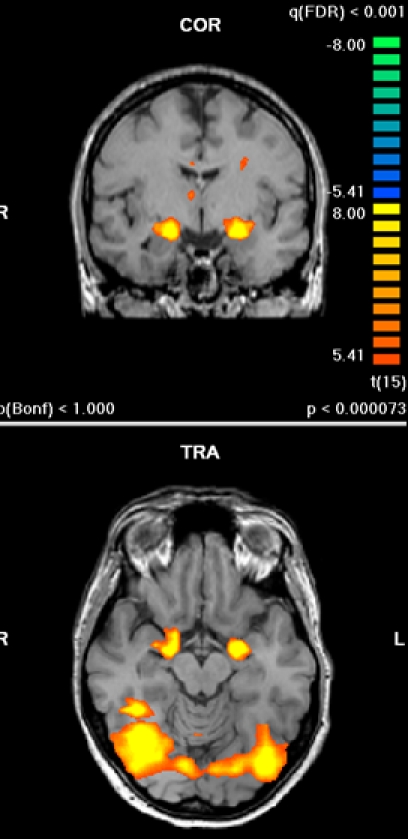
Bilateral activation of amygdala during perception of contempt and disgust expressions (shared activation; conjunction analysis).

Subjects on average identified the target correctly on 98.7% of trials, with 1.4% of false alarms. Across all participants, facial expressions of contempt and disgust (relative to random dot patterns) activated a network of brain regions that included prefrontal areas (superior, middle and medial prefrontal gyrus), anterior cingulate, insula, amygdala, parietal cortex, fusiform gyrus, occipital cortex and putamen and thalamus. Neutral expressions also activated these areas, with exception of insula and cingulate/prefrontal areas. The different types of expressions did not differ in terms of amygdala activation, which was robust and bilateral (see figure 1 for the shared amygdala activation during contempt and disgust as revealed by a conjunction analysis). The amygdala activation also survived a higher threshold of k = 20 and FDR correction (at *P*<0.001). Because of the strong a priori hypothesis regarding preferential amygdala activation for contempt, we conducted an additional analysis in which we lowered the threshold to a liberal value of *P* = 0.005, uncorrected. Contrary to the hypothesis, the contrast of contempt versus disgust yielded significant stronger amygdala activation for disgust relative to contempt, 7 voxels, peak t-value 4.06, peak coordinates 31, −3, −11.

**Figure 2:**
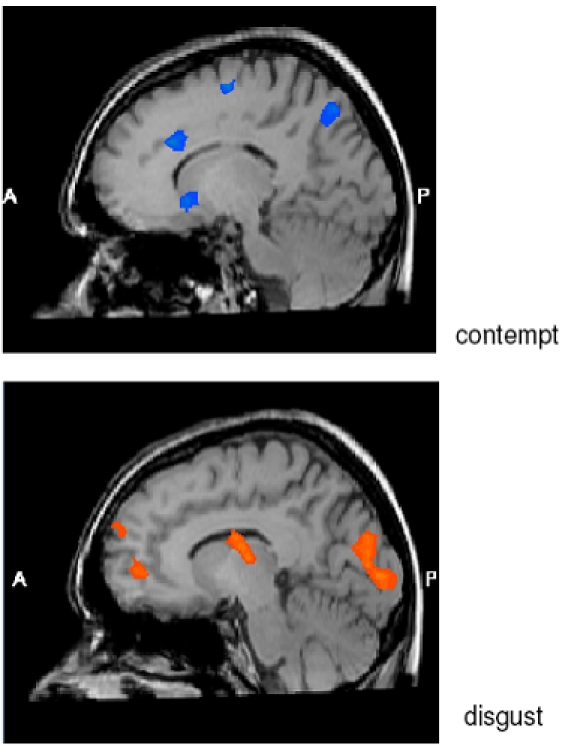
Brain regions activated stronger in men than in women (blue color) or stronger in women than in men (orange color) in response to emotional expressions of contempt (above) or disgust (below).

When men and women were contrasted in a group comparison, a striking difference emerged in activation to contemptuous faces, which was much stronger in men than in women. Contemptuous facial expressions elicited stronger activation in men than in women across a range of brain regions, including the medial frontal gyrus and caudate, left superior temporal gyrus, left inferior frontal gyrus, left superior and inferior parietal lobule, right superior and middle occipital gyrus, and right precuneus. Conversely, for disgusted faces, activation was much stronger in women than in men. This was the case for a large number of clusters, with the most prominent differences in the medial and superior frontal gyrus, right precentral gyrus, left cuneus, right subgyral cortex, right superior temporal gyrus, and bilateral parahippocampal gyrus, insula and thalamus. Table 1 provides coordinates, peak t-values and cluster sizes for differentially activated regions in men compared to women for facial expressions of contempt and disgust, respectively (relative to standards). Figure 2 illustrates several regions that activated differentially for men and women during contempt and disgust perception, respectively.

**Table 1:** Brain regions that were significantly more active in men than women (first part of table) or more active in women than men (second part) for emotional expressions of contempt or disgust (relative to standards; columns indicate hemisphere, talairach coordinates [x, y, z], peak t-value and number of active voxels in the cluster).

men>women		Contempt					Disgust				
		*x*	*y*	*z*	*t*	*voxels*	*x*	*y*	*z*	*t*	*voxels*
Inferior Frontal Gyrus	L	−45	2	31	5,67	71					
Medial Frontal Gyrus	R	24	35	22	5,20	17					
	L	−12	26	28	4,82	21					
Superior Temporal Gyrus	L	−51	2	1	4,98	26					

women>men		Contempt					Disgust				
		*x*	*y*	*z*	*t*	*voxels*	*x*	*y*	*z*	*t*	*voxels*
Inferior Frontal Gyrus	L						−36	17	−14	3,46	10
Medial Frontal Gyrus	L						−12	50	4	4,48	14
Middle Frontal Gyrus	L						−33	8	56	4,42	12
Precentral Gyrus	R						36	−28	58	4,29	14
Superior Frontal Gyrus	R						6	62	28	4,64	98
	R						18	29	53	5,81	48
Superior Temporal Gyrus	R						60	−13	−2	3,90	13
Parahippocampal Gyrus	R						21	−55	−2	6,10	14
	L						−21	−34	−2	4,60	21
Insula	R						30	−28	13	3,13	
	L						−30	−16	25	4,06	46

**Figure 3:**
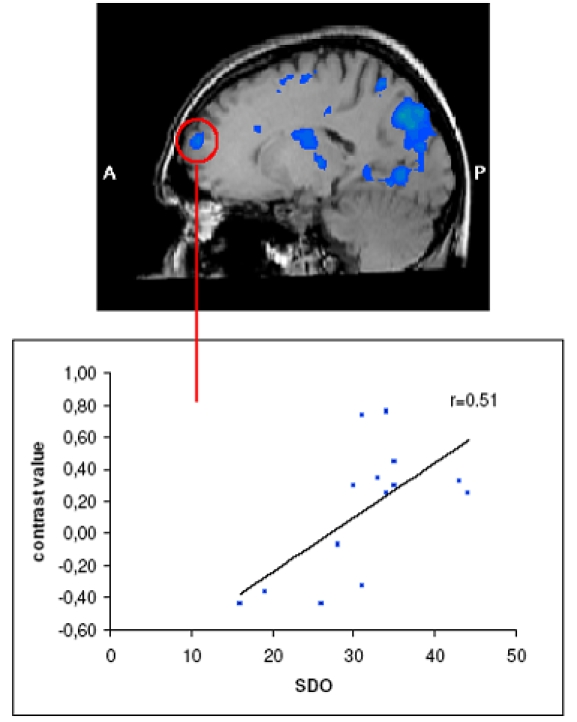
Brain regions activated stronger in men than in women for the contrast of contempt – disgust (there were no regions that were activated stronger in women for this contrast). Activation in the left superior frontal gyrus was associated with scores on the Social Dominance Orientation scale.

We also performed a direct contrast of activation during perception of contemptuous versus disgusted faces. Regions that were more active in men than in women concerned the left superior and middle frontal gyrus, the right inferior and middle frontal gyrus, the right superior temporal gyrus and inferior parietal lobe, the right precuneus, the left precentral gyrus and the cingulate gyrus (see table 2). A region-of-interest analysis of the activated regions that differentiated between men and women revealed a significant positive correlation between activation of the left superior frontal gyrus and ratings on the Social Dominance Orientation Scale [10], r = 0.51, p = 0.05 (see figure 3).

**Table 2:** Brain regions that were significantly more active in men than women (there were no areas more active in women than men) for the contrast of contempt versus disgust (talaraich coordinates, peak t-value and number of active voxels in the cluster).

men>women		*x*	*y*	*z*	*t*	*voxels*
Superior Temporal Gyrus	R	62	−9	3	4.69	27
Inferior Frontal Gyrus	R	43	8	32	4.96	64
Middle Frontal Gyrus	R	28	1	56	4.99	57
Middle Frontal Gyrus	R	29	52	19	4.93	69
Cingulate Gyrus	R	7	30	26	4.38	10
Middle Frontal Gyrus	L	−13	−9	59	5.26	15
Medial Frontal Gyrus	L	−13	6	55	3.72	19
Superior Frontal Gyrus	L	−15	63	19	4.61	24
Precentral Gyrus	L	−27	−14	58	4.03	23
Middle Frontal Gyrus	L	−27	42	34	6.48	20
Precentral Gyrus	L	−40	−12	35	5.56	176
Superior Temporal Gyrus	L	−56	−3	4	3.94	27

Table 3 shows sex differences in activation to male versus female faces. This analysis primarily showed that, for women, contemptuous male faces elicited more brain activation, with significant clusters in the insula, bilaterally, and right middle frontal gyrus. For disgust, men displayed more activation to male as compared to female faces in the left claustrum/insula.

**Table 3:** Brain regions that were significantly more active in men and in women, respectively, for male faces denoting disgust or contempt (as compared to female faces denoting the same emotion).

		*x*	*y*	*z*	*t*	*voxels*
**male: disgust male- female**						
Claustrum/insula	L	−27	17	−4	7,05	12
**female: contempt male- female**						
middle frontal gyrus	R	28	37	30	6,17	13
insula	L	−47	−23	23	8,01	36
insula	R	31	19	5	6,37	15

Contrasts that are not mentioned in the table did not show significant activation differences.

The target, i.e. the neutral face the subject reacted to with a button press during the oddball task, activated (relative to neutral distractors) bilateral superior frontal gyrus, left anterior cingulate gyrus and medial prefrontal cortex, right superior parietal lobule, right middle and superior temporal gyrus, right precentral and postcentral gyrus and right occipital cortex. There were no strong sex differences in response to the target face, with three small regions more activated in women (right prefrontal, precentral gyrus and left posterior parietal).

## Discussion

The aim of this study was to investigate the neural correlates of perceiving facial expressions of contempt and disgust, with a particular interest in sex differences. Specifically we assessed (1) whether the amygdala would be preferentially activated in response to facial expressions of contempt, (2) whether men would show stronger activation than women for expressions of contempt, but not of disgust, and (3) whether there would be gender differences in perceiving same-sex versus opposite-sex contemptuous faces. We used an oddball paradigm, in which participants reacted to a neutral target face, but were also forced to pay attention to the distractor faces with expressions of contempt and disgust. In this way, we could study automatic activation to these emotional expressions without requiring conscious evaluative processing of the particular emotions. The activation of dorsal parietal, cingulate and prefrontal cortices to the target is consistent with previous oddball studies using fMRI [11], [12]. Our primary interest, however, concerned the expressions of contempt and disgust.

Key areas that were activated during perception of contemptuous and disgusted faces included prefrontal areas, anterior cingulate, insula, amygdala, parietal cortex, fusiform gyrus, putamen and thalamus. Whereas cognitive tasks with emotional stimuli tend to activate the left amygdala [13], our attentional viewing task yielded robust bilateral amygdala activation. The only previous fMRI study of expressions of contempt and disgust [7] also reported activation of amygdala, anterior cingulate and putamen for contempt and amygdala, insula, and prefrontal areas for disgust. However, in that study contempt was only contrasted with facial expressions of disgust and with neutral faces. A limitation of this approach is that neutral faces also activate emotional brain regions, notably the amygdala, superior temporal cortex, fusiform gyrus and inferior prefrontal areas [14], [15]. Our approach of contrasting the face stimuli with the standards (random dot patterns) warrants higher sensitivity in evaluating neural responses to faces, which are socio-emotional stimuli by definition.

In contrast to Sambataro et al. [7], we did not find stronger amygdala activation to comtemptuous faces than to disgusted faces. Although we used the same stimulus set, this difference could be due to other differences in the task and study design. Whereas our task only required attention to the faces, the task used by Sambataro et al. required a gender judgment, and thus an explicit evaluation of each face. Lange et al. [16] reported stronger limbic activation to passive viewing of emotional facial expressions as compared to a gender-decision task. Winston et al. [17] found amygdala activation to be strongest for high intensity emotional expressions. The disgust expressions we used are typically rated by subjects to have a higher intensity than the contempt expressions [7], which may explain the stronger amygdala activation for disgust we observed at a liberal threshold. The lack of amygdala activation for disgust expressions in the study by Sambataro et al. [7] may be due to subtracting from other face conditions and to habituation of the amygdala in the block design with repetitive presentation of only face-stimuli. The fact that they did find some amygdala activation for contempt expressions relative to disgust might be explained by a novelty-effect, as the contempt expression is much less common than disgust [5].

We observed a marked dissociation between men and women in activation patterns to contempt and disgust expressions. Stronger activation in women as compared to men for disgust expressions corroborates and extends previous findings from an fMRI study using disgusting stimuli [18]. Whereas Caseras et al. [18] used pictures of disgusting scenes (taken from the International Affective Picture System), we used facial expressions of disgust. Our finding of stronger activation in men for contempt expressions is novel, however. As contempt signals superiority and interpersonal hierarchy, our results may imply a neural basis for higher sensitivity to such signals in men than in women. Indeed, a body of research in the social sciences has found men in general to be more competitive than women and more sensitive to issues of social and hierarchical ranking [9], [19]–[21]. Thus, our finding may parallel the results of an fMRI study in which stronger neural responses were observed in men to scenes of violence and aggression [22].

As to the areas that were activated stronger in men than in women during perception of contemptuous faces, a number of regions has been implicated in emotion processing before. For example, involvement of lateral prefrontal cortex has been implied in emotion regulation [23], [24]. Interestingly, a recent study reported men to activate superior and middle frontal cortex more than women during successful encoding of neutral faces [25]. Our intepretation that the stronger activation in men than women to contempt may reflect higher sensitivity in men to facial expressions denoting social hierarchy is consistent with previous neuroimaging studies that showed a stronger activation in men relative to women in both medial and inferior frontal gyri [26], as well as occipital areas [26], [27] when viewing pictures from categories to which men are generally more sensitive than women (sports, erotica). Activation of the left medial prefrontal cortex has been reported for socio-normative moral judgments relative to grammatical judgments [28], although the activated area was more ventral than the medial prefrontal area in our study. With regard to the caudate, Shirao et al. [29] observed that it was activated stronger in women while perceiving unpleasant linguistic stimuli concerning interpersonal relationships. Our finding of stronger activation in men while perceiving facial expressions of contempt may reflect a visuospatial and hierarchical counterpart of women's sensitivity to verbal expressions of interpersonal conflict.

Our finding of a correlation between activation of the left superior frontal gyrus and ratings on the Social Dominance Orientation Scale is of considerable interest. This area has been shown to activate in previous fMRI studies in response to aversive facial expressions [30] and, also using an oddball paradigm, to sad pictures, including facial expressions or scenes of humans crying [11]. In addition, the superior frontal gyrus has been implicated in the explicit evaluation of facial emotional expressions [31], but has also in emotion regulation [32]. Thus, a higher sensitivity in men for faces expressing contempt could induce stronger emotion regulation efforts, mediated by the superior frontal gyrus.

Notably, stimulus sex had different effects on activation patterns in women and men. Specifically, women showed stronger activation of insula and middle frontal gyrus to male expressions of contempt (as compared to female expressions). Thus, women activate regions that have been associated with processing of aversive stimuli [18]. The stronger reaction in women to male than to female expressions of contempt may have relevance for understanding gender differences in relationships. Expression of contempt has been shown to play a role in conflict in romantic relationships [33], and gender-specific reactions to other-sex contempt expressions may ultimately shed more light on processes that might underlie appraisals of the others' affective state and intentions in relationships [34]. On the basis of the dominance model and intra-sexual competition, it could be hypothesized that men should react especially strongly to contempt on a male face. However, we did not find evidence for this.

Both men and women searched for a male target face. A possible interpretation of our findings of strong sex differences in brain activation could be that men and women simply use different strategies to perform the target detection task. Indeed, it could be argued that for men, the task to search for a male face elicits different neural activity than for women that search for a male face. However, the direct comparison of men and women on activation for the targets does not support this as an alternative explanation for our findings regarding contempt and disgust.

In conclusion, we found evidence of a stronger brain activation in men compared to women in response to faces denoting interpersonal superiority. The fact that men have been shown to have significantly higher social dominance scores than women even after controlling for demographic, and situational factors such as age, social class, religion, educational level, political ideology, ethnicity, racism, region of national origin, and gender-role relevant opinion [9], suggests a universal biological basis. At a more individual level, dominance has been related to testosterone levels [35]. Our study was also confined to stimuli representing individuals (rather than social groups). As sensitivity to interpersonal and intergroup dominance may share underlying mechanisms, our findings may be a first step towards a neural basis for sex differences in sensitivity to social hierarchy and dominance.

## Materials and Methods

Sixteen healthy subjects (8 men, 8 women) participated in the study after providing written informed consent. Approval was obtained from the institutional ethics board, METC UMCG, for the use of human participants in this study. Mean age was 22.5 years (SD = 2.5). Subjects confirmed that they did not have a history of seeking or receiving treatment for any neurological or psychiatric disorder. Men and women did not differ on positive and negative affect as measured with the Positive and Negative Affect Scale [36].

### Task

Participants viewed pictures of faces from a standard set [37], depicting expressions of contempt and disgust in addition to neutral expressions. Matsumoto and Ekman [13] demonstrated that subjects reliably associated the contempt expressions in this set with situations that elicit contempt. Disgust was included as a control emotional expression in our study because it is conceptually close to contempt [1] and it is the emotion subjects most often confound contempt with [5]. In contrast to contempt, however, disgust is not by definition inherently interpersonal and it is not by definition related to social hierachy. Therefore, we selected disgust as the ideal comparison emotional expression.

**Figure 4:**
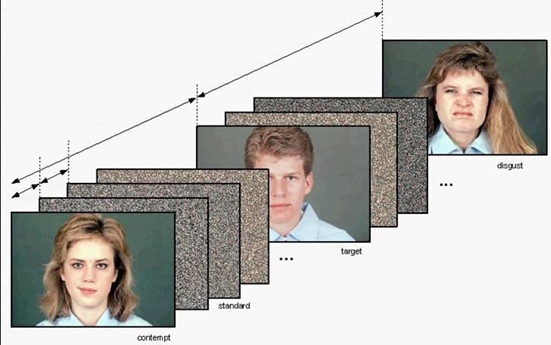
Presentation of stimuli in the visual oddball task with emotional faces as distractors and random dot patterns as standards. All stimuli (faces and standards) were presented for 1.9 s. There were 9, 10 or 11 standards after each face presentation (this was randomized). Total duration of the task was approximately 35 min.

The task was analogous to the visual oddbal task described by Wang et al. [11], who reported activation of emotional brain areas using such a design with affective pictures. The task was scanned in an event-related design in which the face-stimuli were presented in a pseudo-random fashion (figure 4). The pictures of the emotional faces were presented along with neutral faces as intermittent task-irrelevant distractors during a concurrent visual oddbal task. Faces were shown for 1.9 s. The subjects were presented with random dot patterns created using the face stimuli (“standards”) interspersed with target stimuli (to which they had to react with a button press) and novel distractors (the actual stimuli of interest for our analyses). Thus, the novel distractors consisted of faces expressing disgust or contempt, or neutral faces. The standards were presented for 82% of trials, whereas the other stimuli (target, neutral faces, emotional faces) were presented for 2.33% of trials. There were 9, 10 or 11 standards after each face presentation (this was randomized). The use of standards in an visual odd-ball task may help prevent emotional brain areas from habituating to emotional stimuli. The task for the subject was to press a response button whenever one specific male face (the target) was presented. In each run there were also three short fixation blocks (8.77% of trials), providing a resting state in which the subject looked at a fixation cross presented in the center of the screen.

### fMRI procedure

The six conditions were presented in three runs (370 stimuli each) in a pseudorandomized design. A 3 T Philips system (Best, The Netherlands) with a sense-8 head coil was used to acquire both T1 anatomical volume images (256×256 matrix, 160 slices, voxel size 1×1 mm; slice thickness 2 mm) and T2* weighted echoplanar images with blood oxygenation level-dependent (BOLD) contrast (64×64 matrix, voxel size 3.5×3.5 mm, TR = 1900 ms, TE = 30 ms, Field of View 224). Each echoplanar image comprised 30 slices (3.5 mm; no gap), positioned to cover the whole brain.

### fMRI Analysis

Data were preprocessed and analyzed using the Brainvoyager QX 1.7 software package (Brain Innovation, Maastricht, the Netherlands). Image preprocessing included: 3D motion correction, slice scan time correction using linear interpolation, spatial smooting with a 6 mm full with at half minimum Gaussian kernel, removal of linear trends, and high pass filter of frequencies below 3 cycles per time course. Spatial normalization was performed using the standard 9-parameter landmark method of Talairach and Tournoux [38].

A general linear model [39] was defined for each subject that included five regressors (target, disgust, contempt, neutral, fillers) which modeled the BOLD response to the epochs following the different stimuli. Each regressor was convolved with a standard gamma model [40] of the hemodynamic impulse-response function. Task-related activity was estimated at a group level using contrasts (e.g. contempt versus fillers or contempt versus disgust) in random effects analyses as implemented in Brainvoyager QX 1.7, thresholded at *P*<0.001, uncorrected, with a cluster threshold of 10 voxels (cf. ref. 41). To evaluate the robustness of amygdala activation, a conjunction analysis of both emotional facial expressions (contempt and disgust) relative to standards was conducted, as implemented in BrainVoyager. In this conjunction analysis, an effect is considered significant only if all the involved contrasts are simultaneously significant. For the (second-level) comparison of men versus women, statistical maps were thresholded for significance *P*<0.01, uncorrected, and cluster size ≥10 voxels. Similar thresholds have previously been used in fMRI investigations of sex differences in neural activation [42], [43]. We limited our analyses to regions in the frontal and temporal lobes, as these have been shown to be most relevant to emotional and social processing [44]. In this way, we reduced the number of statistical comparisons.

To investigate whether sex differences in brain activation would be associated with social dominance scores, we defined functional regions-of-interest (ROI's) based on the activated regions that differentiated between men and women in the contempt vs. disgust contrast. Mean β-values were correlated to scores on the Social Dominance Orientation Scale [10].
